# Protective effect of miRNA-containing extracellular vesicles derived from mesenchymal stromal cells of old rats on renal function in chronic kidney disease

**DOI:** 10.1186/s13287-020-01792-7

**Published:** 2020-07-08

**Authors:** Yan Wang, Yi Fang Guo, Guang Ping Fu, Chang Guan, Xin Zhang, Dong Gang Yang, Yun Cong Shi

**Affiliations:** 1 Department of Geriatric Cardiology, Hebei General Hospital, Shijiazhuang, Hebei China; 2 Hebei Key Laboratory of Forensic Medicine, Department of Forensic Medical, Hebei Medical University, Shijiazhuang, Hebei China; 3 Hebei Medical University, Shijiazhuang, Hebei China; 4 Northern College, Zhangjiakou, Hebei China

**Keywords:** Extracellular vesicle, Mesenchymal stromal cell, miRNAs, Old, Chronic kidney disease

## Abstract

**Introduction:**

Mesenchymal stromal cells (MSCs) play an important role in the prevention of cell and tissue fibrosis. Senescence may decrease the function of MSCs during recovery from tissue and organ damage. Extracellular vesicles (EVs) released from MSCs contribute to the repair of kidney injury. We explored the influence of senescence on EVs derived from MSCs (MSC-EVs) and detected the protective effects of MSC-EVs expressing low levels of miR-294/miR-133 derived from old rats against chronic kidney disease (CKD).

**Methods:**

The effects of MSC-EVs derived from 3-month-old and 18-month-old male Fisher 344 rats on renal fibrosis were explored in a unilateral ureteral obstruction (UUO) model. pLV-miR-294/pLV-miR-133 mimic/inhibitor were injected into young and old rats before UUO to detect the effects of miR-294/miR-133, which were decreased in MSC-EVs and sera from old rats, on renal function in CKD. Transforming growth factor-β1 (TGF-β1)-induced human renal proximal tubular epithelial (HK2) cells were used to imitate the pathological process of renal fibrosis in vitro. Western blotting was used to assess the expression of epithelial/mesenchymal markers and phosphorylation of proteins in HK2 cells.

**Results:**

The inhibition of UUO-induced CKD by MSC-EVs was weaker in old rats than in young rats. Downregulation of miRNAs (miR-294 and miR-133) in both MSC-EVs and sera from old rats obviously attenuated UUO-induced renal injury in old rats. miR-294 and miR-133 overexpression mitigated TGF-β1-mediated epithelial-mesenchymal transition (EMT) in HK2 cells, and the obvious increase in the phosphorylation of both SMAD2/3 and ERK1/2 induced by TGF-β1 was prevented in miR-294- and miR-133-overexpressing HK2 cells.

**Conclusions:**

The ability of MSC-EVs to inhibit renal fibrosis decreased with age. miR-294/miR-133 in MSC-EVs and sera had an important effect on renal fibrosis in old rats and on EMT in HK2 cells. Furthermore, miR-294/miR-133 overexpression prevented SMAD2/3 and ERK1/2 phosphorylation in HK2 cells during TGF-β1-mediated EMT. These findings show that miR-294/miR-133 may be therapeutic in renal fibrosis and related renal dysfunction in elderly individuals.

## Background

Chronic kidney disease (CKD) has a high morbidity in elderly people, and a considerable number of CKD patients progress to end-stage renal disease (ESRD). Renal fibrosis, the main pathological process of CKD, is characterised by extracellular matrix (ECM) accumulation [1]. Current therapeutic modalities for CKD are limited, and there are no efficacious therapies to repair renal damage [2]. Nevertheless, over the past decade, interest in mesenchymal stromal cells (MSCs) as a novel regenerative therapy for renal injury has increased [3]. MSCs are pluripotent stem cells that can differentiate into cells of several lineages [4]. Recent studies have shown that extracellular vesicles (EVs) derived from MSCs (MSC-EVs), an important paracrine medium of MSCs, contain mRNAs and microRNAs (miRNAs) that induce genetic and epigenetic changes in target cells, improving renal outcomes in CKD [5].

Unfortunately, the function of MSCs is known to decline with age, which may result in a reduction in the maintenance of tissue homeostasis leading to organ failure and diseases of ageing [6]. The proliferative activity of MSCs also declines during the process of ageing, decreasing its treatment effects on organ disease. Our previous study found that MSCs derived from old rats became senescent with age. Further assessment of the effects of MSC-EVs from old rats showed an obvious decline in their ability to prevent transforming growth factor-β1 (TGF-β1)-mediated epithelial-mesenchymal transition (EMT) of renal HK2 cells. Microarray analysis of miRNAs in MSC-EVs from young and old rats indicated that many miRNAs were differentially expressed and that many of these changes in expression were significant. Consistent with the downregulated miRNA expression observed in old MSC-EVs, serum analysis showed significantly decreased expression of miR-294 and miR-133 in old rats [7].

The incidence of various diseases in the elderly gradually increases with age. The physiological characteristics of the elderly are different from those of young individuals, and the elderly exhibit a widespread decrease in the physiological functions of many organs and MSCs due to the senescence process [8]. However, no studies have assessed the influence of senescence on the effects of MSC-EVs on renal function. In this study, we aimed to observe the inhibition of unilateral ureteral obstruction (UUO)-induced CKD mediated by MSC-EVs from old rats and assess the protective roles of miR-294/miR-133, which were downregulated in both the sera and MSC-EVs of aged rats, in preventing CKD to further explore a possible mechanism to mitigate renal fibrosis. Our results provide a new theoretical basis for MSC-related gene therapy for CKD in elderly people.

## Methods

### Isolation and identification of young and old MSCs

Animal welfare was ensured, and all experimental procedures were carried out in accordance with the Guide for the Care and Use of Laboratory Animals (Ministry of Science and Technology of China, 2006); experimental protocols were approved by the Animal Ethics Committee of Hebei General Hospital (No. 202009). Bone marrow mesenchymal stromal cells (BM-MSCs) were extracted from 3-month-old and 18-month-old male Fisher 344 rats and cultured in specific MSC medium (Cyagen Bioscience Inc., Santa Clara, CA, USA) for the P2 generation. Then, purified P2-MSCs at 10^5^ cells/well were grown on six-well culture plates and then identified according to their osteogenic/adipocyte differentiation by Alizarin Red S and Oil Red O staining as previously described [7]. The MSC surface markers CD44, CD45 and CD90 (BioLegend, Inc., USA) were also detected using flow cytometry.

### Extraction and identification of young and old MSC-EVs

Cells were starved overnight in serum-free medium supplemented with 0.5% bovine serum albumin (Sigma-Aldrich, USA) prior to the collection of EVs as previously described [9]. The MSC-EVs were extracted from the supernatant of 3 × 10^5^ P2 generation MSCs after overnight incubation (with cellular debris removed from the supernatant by centrifugation at 2000×*g* for 20 min) using an exoEasy Maxi Kit (Qiagen, Germany) according to the manufacturer’s directions.

The collected EVs were resuspended in phosphate-buffered saline (PBS) containing phycoerythrin (PE)- or fluorescein isothiocyanate (FITC)-labelled anti-CD29, anti-CD44 and anti-alpha 4-integrin antibodies (BioLegend, Inc., USA). Rat IgG labelled with PE or FITC (BioLegend, Inc., USA) was used as a negative control. The MSC-EV surface markers CD44, CD29 and alpha 4-integrin were detected by flow cytometry.

### Lentivirus miRNA construct and administration time

Recombinant lentiviral vectors expressing rno-miR-294 inhibitor/rno-miR-294 mimics/rno-miR-133 inhibitor/rno-miR-133 mimics (LV-miRNA) and the corresponding control (LV-NC) were constructed as described previously [10]. Lentiviruses were prepared by cotransfection of the lentiviral constructs and packaging vectors into 293 T cells (American Type Culture Collection, Manassas, VA, USA) using RNAi-Mate (GenePharma, Inc., Shanghai, China). Virus-containing supernatants were collected 72 h after infection. Viruses were recovered by ultracentrifugation (20,000 rpm at 4 °C for 2 h) and resuspended in PBS. Viral titres were determined by infecting 293 T cells with serially diluted concentrated lentiviral preparations.

The sequences of the miRNA mimics or inhibitors are as follows:

- rno-miR-294 inhibitor, 5′-AGATAGGGCCTCCATTTTGAG-3′;
- rno-miR-294 mimics, 5′- CTCAAAATGGAGGCCCTATCT-3′;
- rno-miR-133b-3p inhibitor, 5′-TAGCTGGTTGAAGGGGACCAAA-3′;
- rno-miR-133b-3p mimics, 5′-TTTGGTCCCCTTCAACCAGCTA-3′

In the preliminary study, the serum from venae angularis were collected from young and old rats at 0, 2 and 3 days after LV-miRNAs mimic/inhibitor injection. As shown in Additional file, the level of miR-294 and miR-133 expression increased/decreased significantly at 3 days after injection, and based on these, we administered inhibitors or mimics of the miRNAs to the rats 3 days before the UUO surgery in this study.

### Induction of kidney injury in rats and treatment

Healthy male-specific pathogen-free (SPF), purpose-bred Fisher 344 rats (3 months and 18 months old) were obtained from the Model Animal Research Center of Hebei General Hospital. Animals had ad libitum access to a rodent diet and tap water. The rats were kept in cages under a 12:12-h light–dark cycle with a temperature of 21 ± 2 °C and a humidity of 55 ± 5%. Young (3 months old) and old (18 months old) rats assigned to UUO modelling were anaesthetized by intraperitoneal injection of sodium pentobarbital and then placed on a heating pad to maintain their body temperature at 37 °C. Their left ureters were ligated with silk (4/0). Amoxicillin was intraperitoneally injected into the peritoneal cavity before it was closed during surgery. The control animals underwent the same procedure, but their ureter was not ligated. The young and old rats were randomly divided into the twelve groups (shown as Table 1). Young rats were injected in the tail vein with Y-MSC-EVs or O-MSC-EVs (3 × 10^5^ P2 generation young/old MSCs released overnight) after surgery. In addition, 200 μl of LVs (10^9^ TU/ml) was injected in the tail veins of young/old rats in these groups at 3 days before UUO surgery. The groups were sacrificed at 7 days and 14 days after UUO surgery. Blood was collected before the rats were sacrificed, and the levels of blood urea nitrogen (BUN), serum creatinine (Scr) and uric acid (UA) were examined using a Beckman Analyser II (Beckman Instruments, Inc., Fullerton, CA, USA).

**Table 1:** The experimental groups of young and old rats

Group	Treatment	Endpoint*
Young control	Control	7 days and 14 days
Old control	Control	7 days and 14 days
Young UUO	UUO	7 days and 14 days after UUO
Old UUO	UUO	7 days and 14 days after UUO
Young-UUO + Y-MSC-EV	Y-MSC-EVs injection with tail vein at day 0 after UUO	7 days and 14 days after UUO
Young-UUO + O-MSC-EV	Y-MSC-EVs injection with tail vein at day 0 after UUO	7 days and 14 days after UUO
Young-UUO + LV-rno-miR-294 inhibitor	LV-rno-miR-294inhibitor injection with tail vein 3 days before UUO	7 days and 14 days after UUO
Young-UUO + LV-rno-miR-133 inhibitor	LV-rno-miR-133inhibitor injection with tail vein 3 days before UUO	7 days and 14 days after UUO
Old-UUO + LV-rno-miR-294 mimic	LV-rno-miR-294 mimic injection with tail vein 3 days before UUO	7 days and 14 days after UUO
Old-UUO + LV-rno-miR-133 mimic	LV-rno-miR-133 mimic injection with tail vein 3 days before UUO	7 days and 14 days after UUO
Young-UUO + LV-NC	LV-NC injection with tail vein 3 days before UUO	7 days and 14 days after UUO
Old-UUO + LV-NC	LV-NC injection with tail vein 3 days before UUO	7 days and 14 days after UUO

Experimental design and the treatment conditions of young and old rats

*Endpoint (either 7 days or 14 days) had 5 rats in each group

### Renal histological and immunostaining analyses

After the rats had been sacrificed, the ligated kidneys were fixed in 4% paraformaldehyde for histological examination. Kidney tissue was embedded in paraffin and cut into 3-μm-thick sections. Standard sequential techniques were used to dewax the sections before haematoxylin-eosin (HE) and Masson’s trichrome (MT) staining. Damage was assessed as described in previous studies [11, 12].

The kidney sections used for immunohistochemical detection were incubated with primary antibodies against α-smooth muscle actin (α-SMA) (1:1000, Cell Signaling Technology, Inc., USA) and E-cadherin (1:1000, Cell Signaling Technology, Inc., USA) according to the manufacturer’s protocols. After washing, the sections were further incubated with anti-rabbit secondary antibody (Abcam, Cambridge, USA) at a 1:5000 dilution. Finally, the sections were counterstained with Mayer’s haematoxylin and dehydrated for further observation. The Graphic context analysis software ImagePro plus 6.0 was used to analyse the immunohistochemistry pictures. Five fields were chosen under a × 200 microscope (OLYMPUS U-LH100-3) for each slice to record the positive staining for the average integral optical density.

### TGF-β1 stimulation and miRNA transfection

HK2 cells (American Type Culture Collection, Manassas, VA, USA) were cultured in 10% foetal bovine serum (Gibco, USA)-containing Dulbecco’s modified Eagle’s medium/F12 (DMEM/F12) complete medium (Corning, USA). When the cells reached 50% confluence, they were synchronised overnight in serum-free DMEM/F12 medium, which was then replaced with complete medium containing 8 ng/ml recombinant human TGF-β1 (PeproTech Inc., USA), before the cells were cultured for 48 h and 72 h to induce fibrosis. miR-294, miR-133 and control miRNA (GeneCopoeia Inc., Rockville, MD, USA) were transfected into synchronised HK2 cells in the miRNA transfection groups with the jet-PRIME® transfection reagent (Polyplus Transfection, Inc., USA) according to the manufacturer’s instructions. After 24 h of transfection, the medium was replaced with complete medium containing 8 ng/ml recombinant human TGF-β1, and the cells were incubated for an additional 48 h and 72 h.

### Western blotting

After proteins were extracted and assessed by protein assay as previously described [7], samples containing 40 μg of protein were subjected to 10% SDS gel electrophoresis, followed by Western blotting using the following primary antibodies:

- Rabbit monoclonal anti E-cadherin (1:1000 dilution; Cell Signaling Technology, Inc., USA),
- Rabbit monoclonal anti-α-SMA (1:1000 dilution; Cell Signaling Technology, Inc., USA),
- Rabbit monoclonal anti-phospho-Smad2/Smad3 (1:1000 dilution; Cell Signaling Technology, Inc., USA),
- Rabbit monoclonal anti-Smad2/3 (1:1000 dilution; Cell Signaling Technology, Inc., USA),
- Rabbit monoclonal anti-phospho-p44/42 MAPK (Erk1/2) (1:2000 dilution; Cell Signaling Technology, Inc., USA),
- Rabbit monoclonal anti-p44/42 MAPK (ERK1/2) (1:2000 dilution; Cell Signaling Technology, Inc., USA), and
- Anti-β-actin (1:4000 dilution; Proteintech Group, Inc., USA).

After washing with primary antibodies, the membranes were incubated with secondary antibodies (1:20,000; Abcam, Cambridge, MA, USA). An enhanced chemiluminescence (ECL) Western blotting kit (Applygen Technologies, Inc., Beijing, China) was used to assess target band expression. β-Actin was used as the internal control, and the relative expression levels of E-cadherin, α-SMA, p-SMAD2/3/SMAD2/3 and phospho-ERK1/2/ERK1/2 in each experimental group were calculated.

### Statistical analysis

The results are expressed as the mean ± standard deviation and were analysed using the SPSS 24.0 software (IBM Corporation, Armonk, NY, USA). Differences between experimental groups were analysed by using one-way analysis of variance. *P* values < 0.05 indicated statistical significance.

## Results

### Characterisation of young/old MSCs and MSC-EVs

**Fig. 1:**
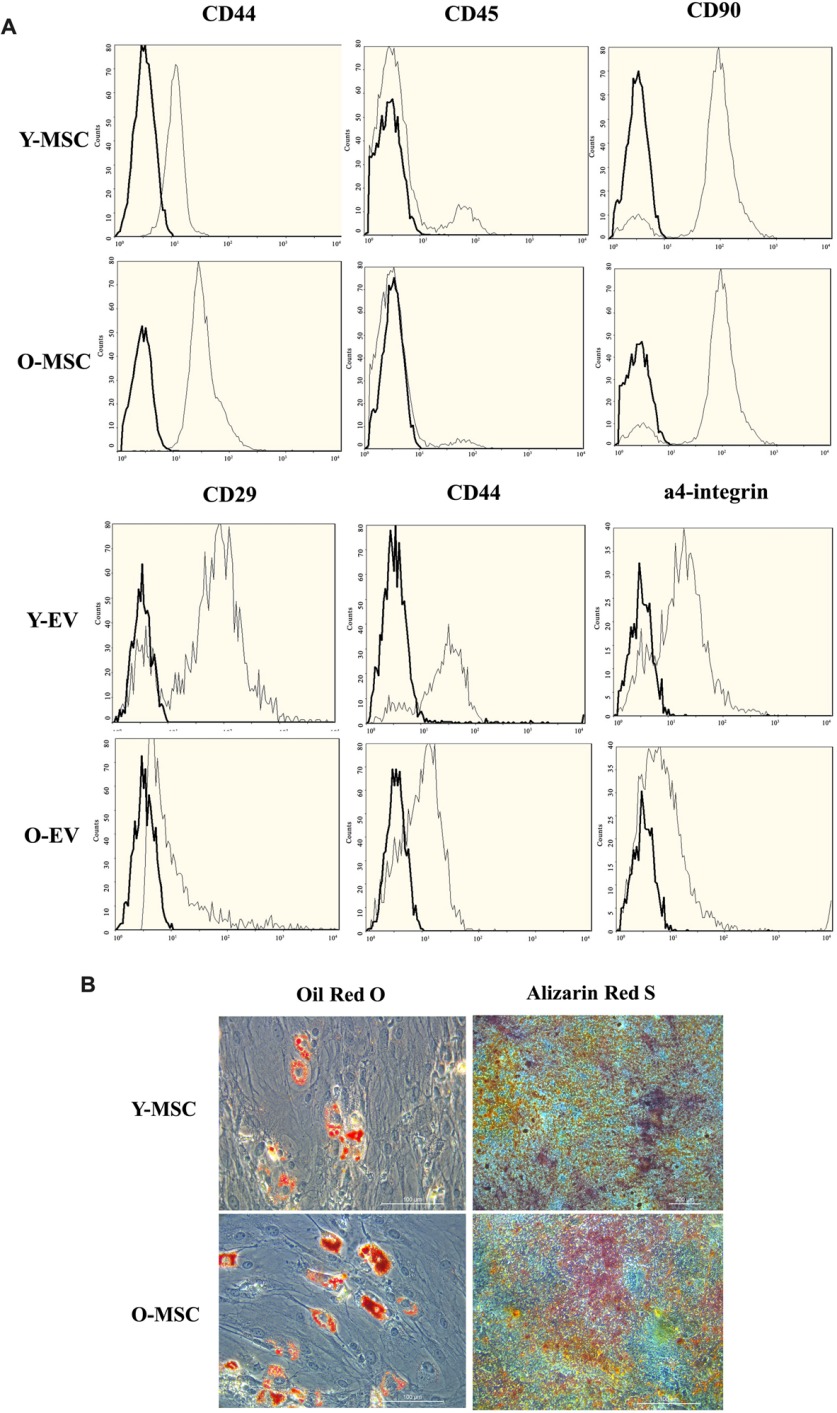
Analysis of the expression of surface markers and characterisation of MSCs and MSC-EVs. **a** Young and old MSCs were labelled with the antibody against CD45, CD44 and CD90; the cells present were positive for CD90 and CD44 but negative for CD45. Representative FACS analyses of young and old MSC-EVs expressed similar results for CD44, CD29 and α4-integrin. **b** Young and old MSCs were cultured in conditions inducive of osteogenic or adipogenic differentiation, respectively. After osteogenic differentiation, calcium in the mineralised extracellular matrix was shown by Alizarin Red S staining. After adipogenic differentiation, lipid droplets were indicated by their staining with Oil Red O

MSCs obtained from the bone marrow of Fisher 344 rats grew into adherent cultures as previously described [7]. Flow cytometry analysis confirmed that the MSCs from both young/old rats were positive for the phenotypic markers CD44 and CD90 and negative for the marker CD45 (Fig. 1a). Expression of the adhesion molecules CD44, CD29 and α4-integrin on the plasma membrane of young/old MSCs was detected (Fig. 1a). MSCs derived from both young and old rats could differentiate into adipocytes and osteoblasts (Fig. 1b).

### Weakened ability of MSC-EVs derived from old rats to inhibit UUO-induced CKD

**Fig. 2:**
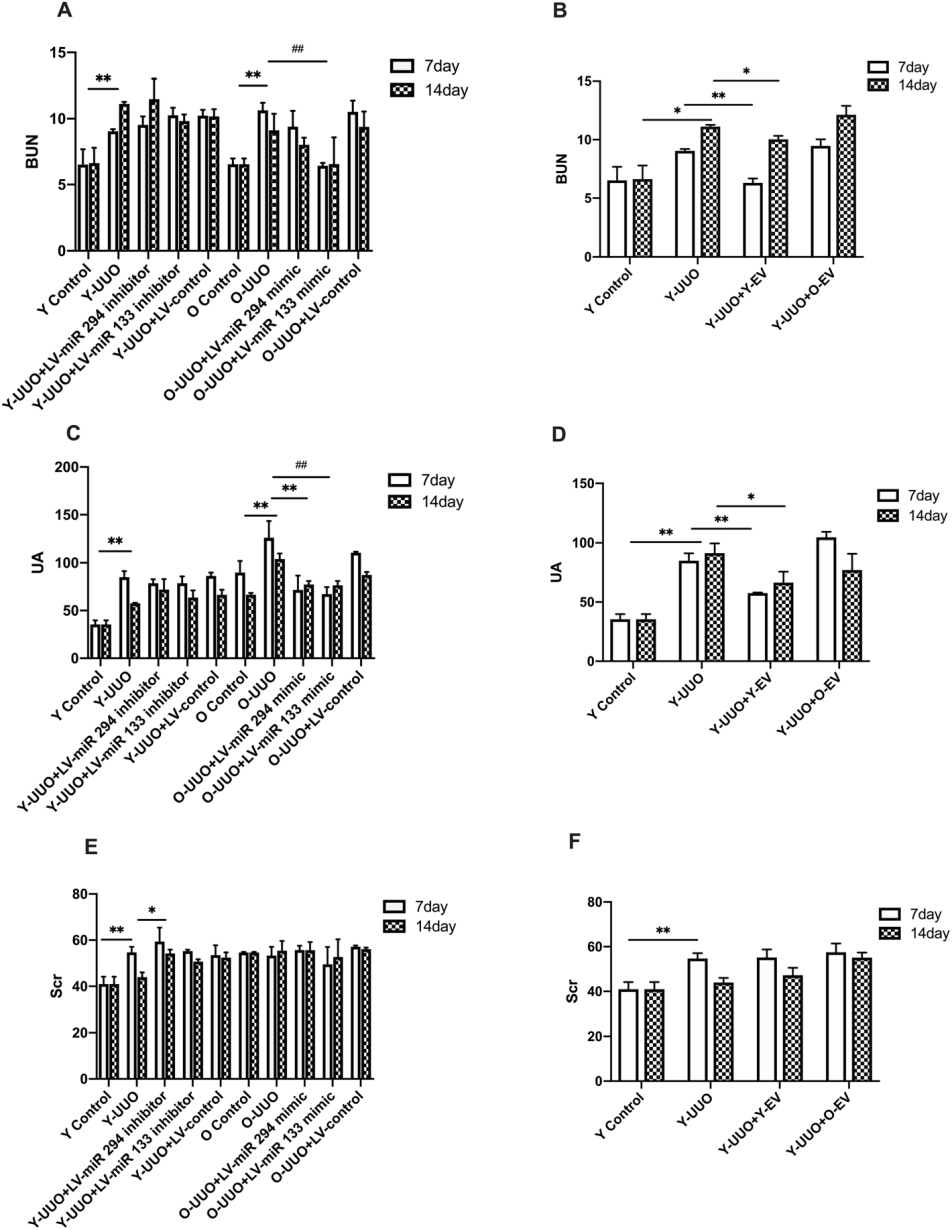
Blood urea nitrogen (BUN), serum uric acid (UA) and serum creatinine (Scr) from different experimental groups. Rats were subjected to unilateral ureteral obstruction (UUO), followed by intravenous injection of young or old MSC-EVs (*n* = 5 each group). BUN (**b**), UA (**d**) and Scr (**f**) were detected at the beginning of the experiments and at an end point of 7 and 14 days after surgery. LVs-miR-294/133 mimic/inhibitors were injected into the tail vein of young/old rats in these groups 3 days before UUO surgery (*n* = 5 each group). BUN (**a**), UA (**c**) and Scr (**e**) were detected at the beginning of the experiments and at an end point of 7 and 14 days after surgery. *p < 0.05, **p < 0.01

**Fig. 3:**
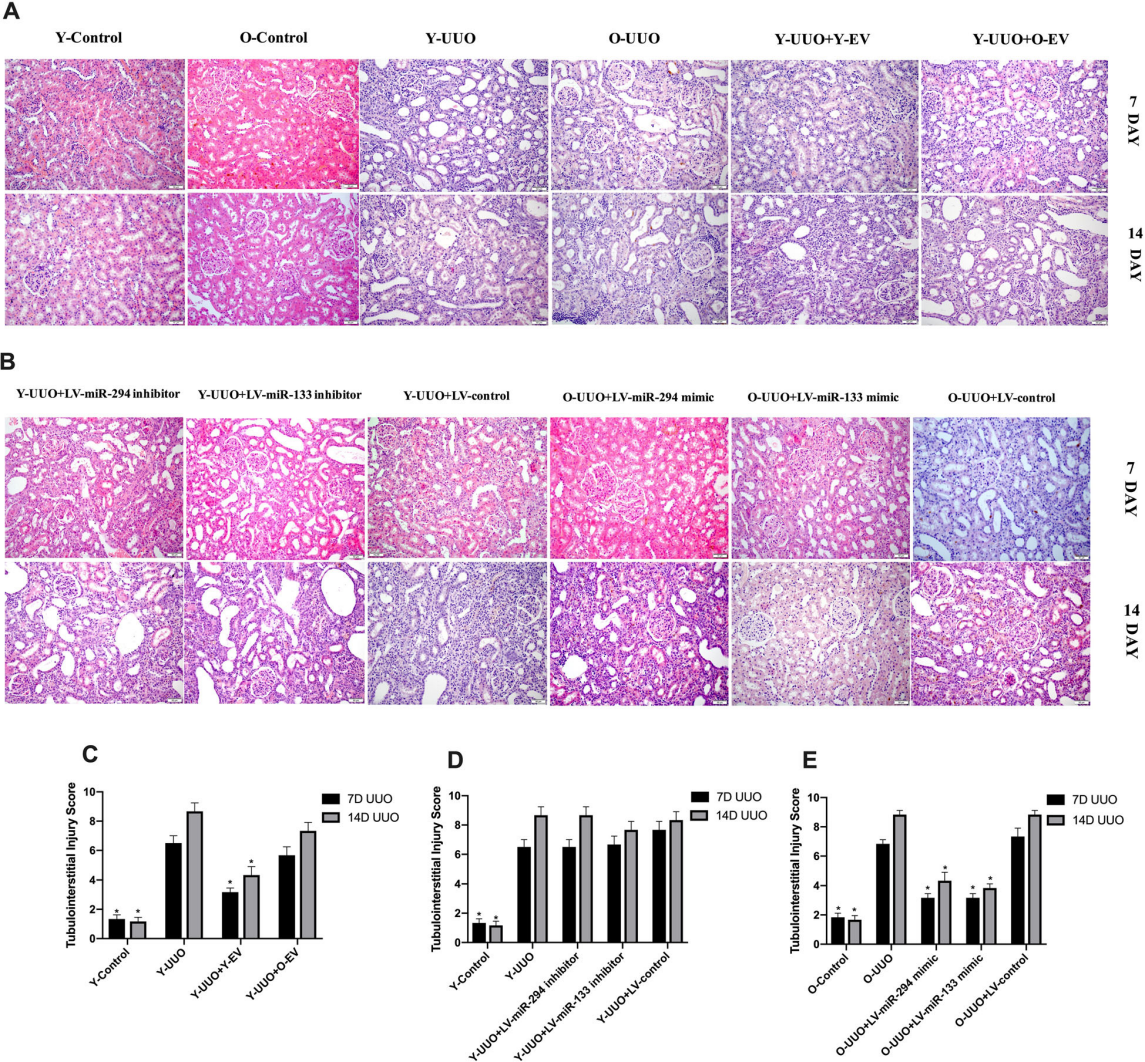
Representative micrographs of tubulointerstitial injury in the obstructed kidney after UUO in different experimental groups (HE staining of rats’ kidney sections, *×* 200). **a** Young and old rats were subjected to unilateral ureteral obstruction (UUO), and HE was used to analyse the histological changes of young or old MSC-EVs treatment at 7 and 14 days after surgery. **b** Young and old rats were subjected to unilateral ureteral obstruction (UUO), and HE was used to analyse the histological changes of LV-miR-294/133 inhibitor/mimic treatment at an end point of 7 and 14 days after surgery. **c**–**e** The quantitative analysis results of tubulointerstitial injury in the kidneys. All data are represented as the mean ± SE. The rat number in each group is 5. *p < 0.05 compared with the UUO group

A significant increase in the levels of BUN and UA was observed on days 7 and 14 after the induction of UUO (Fig. 2b, d). The level of Scr was significantly elevated on day 7 after UUO treatment (Fig. 2f). These changes were associated with histological changes in kidney tissue, including tubular dilation, apoptosis, necrosis and the presence of proteinaceous casts in the tubules (Fig. 3a). However, the tubular lesions were significantly reduced in UUO rats injected with Y-MSC-EVs after surgery compared to UUO rats treated with O-MSC-EVs. UUO rats treated with only Y-MSC-EVs exhibited significantly decreased levels of BUN and UA on days 7 and 14 compared to those of UUO rats treated with O-MSC-EVs, and no change in Scr levels was detected (Fig. 2b, d, f).

**Fig. 4:**
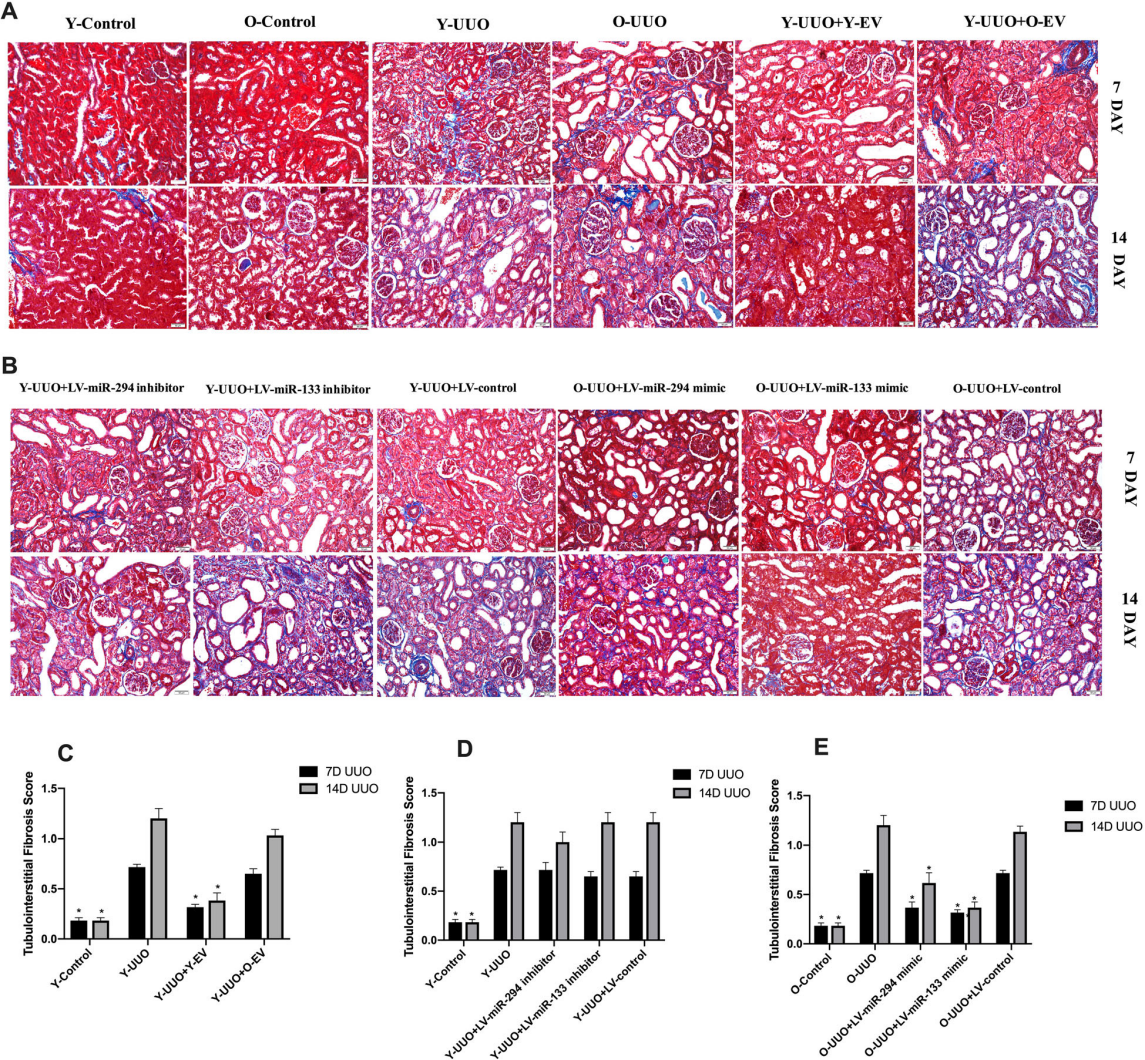
Representative micrographs of collagen expression in rat UUO kidney sections with Masson’s trichrome (MT) staining from different experimental groups (magnification, × 200). **a** Young and old rats were subjected to unilateral ureteral obstruction (UUO), and MT staining was used to analyse the changes in collagen expression of young or old MSC-EVs treatment at 7 and 14 days after surgery. **b** Young and old rats were subjected to unilateral ureteral obstruction (UUO), and MT staining was used to analyse the changes in collagen expression of LV-miR-294/133 inhibitor/mimic treatment at an end point of 7 and 14 days after surgery. **c**–**e** The quantitative analysis of collagen expression in the kidneys. All data are represented as the mean ± SE. The rat number in each group is 5. *p < 0.05 compared with the UUO group

**Fig. 5:**
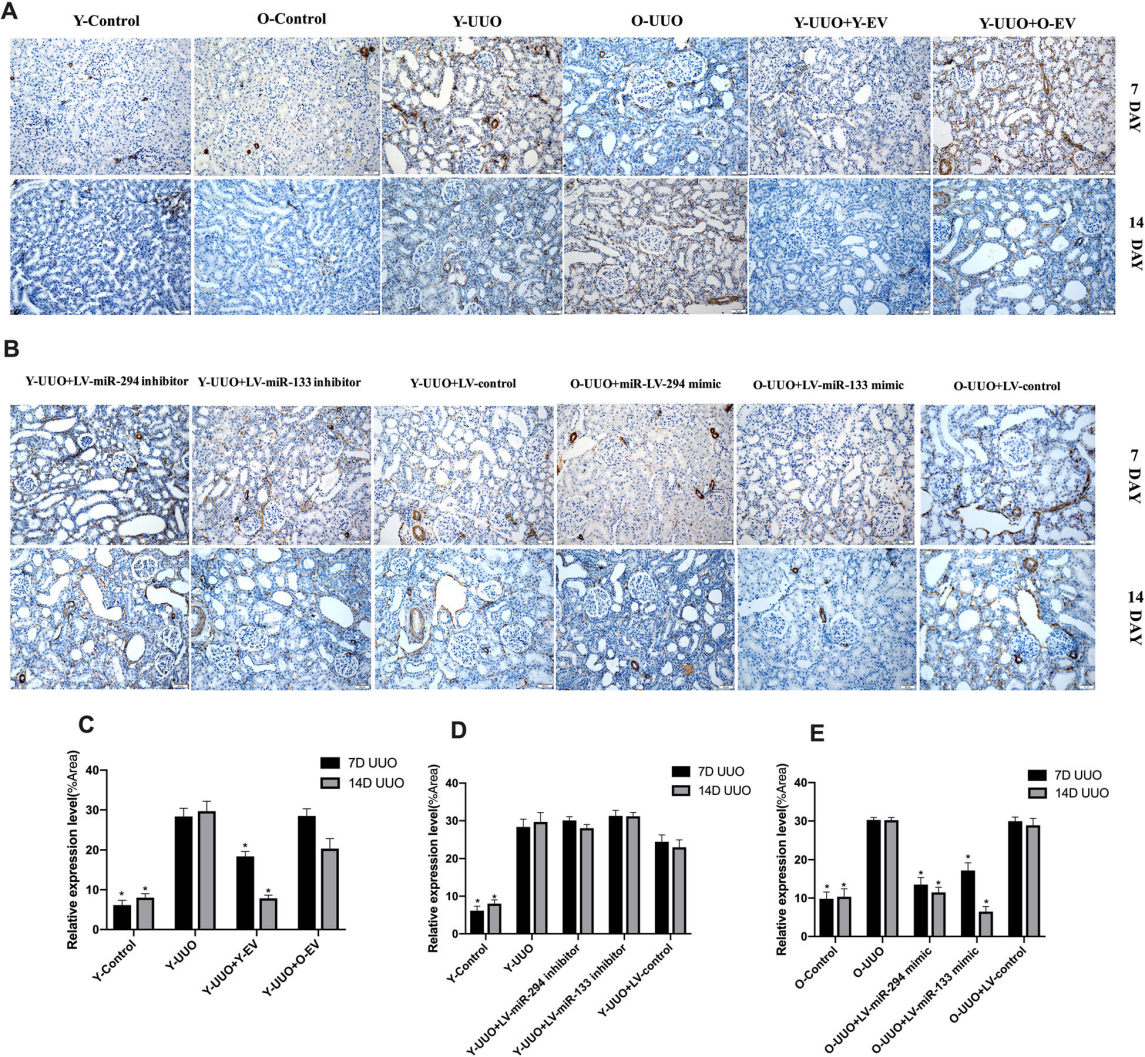
Immunohistochemistry of α-SMA on the renal histology of kidney sections. **a** The expression of α-SMA at 7 and 14 days after surgery in control rats and in UUO rats treated with young and old MSC-EVs. **b** The expression of α-SMA at 7 and 14 days after surgery in control and UUO rats injected with LV-miR-294/133 inhibitor/mimic. **c**–**e** The quantitative analysis of α-SMA expression in the kidneys. All data Are represented as the mean ± SE. The rat number in each group is 5. *p < 0.05 compared with the UUO group

**Fig. 6:**
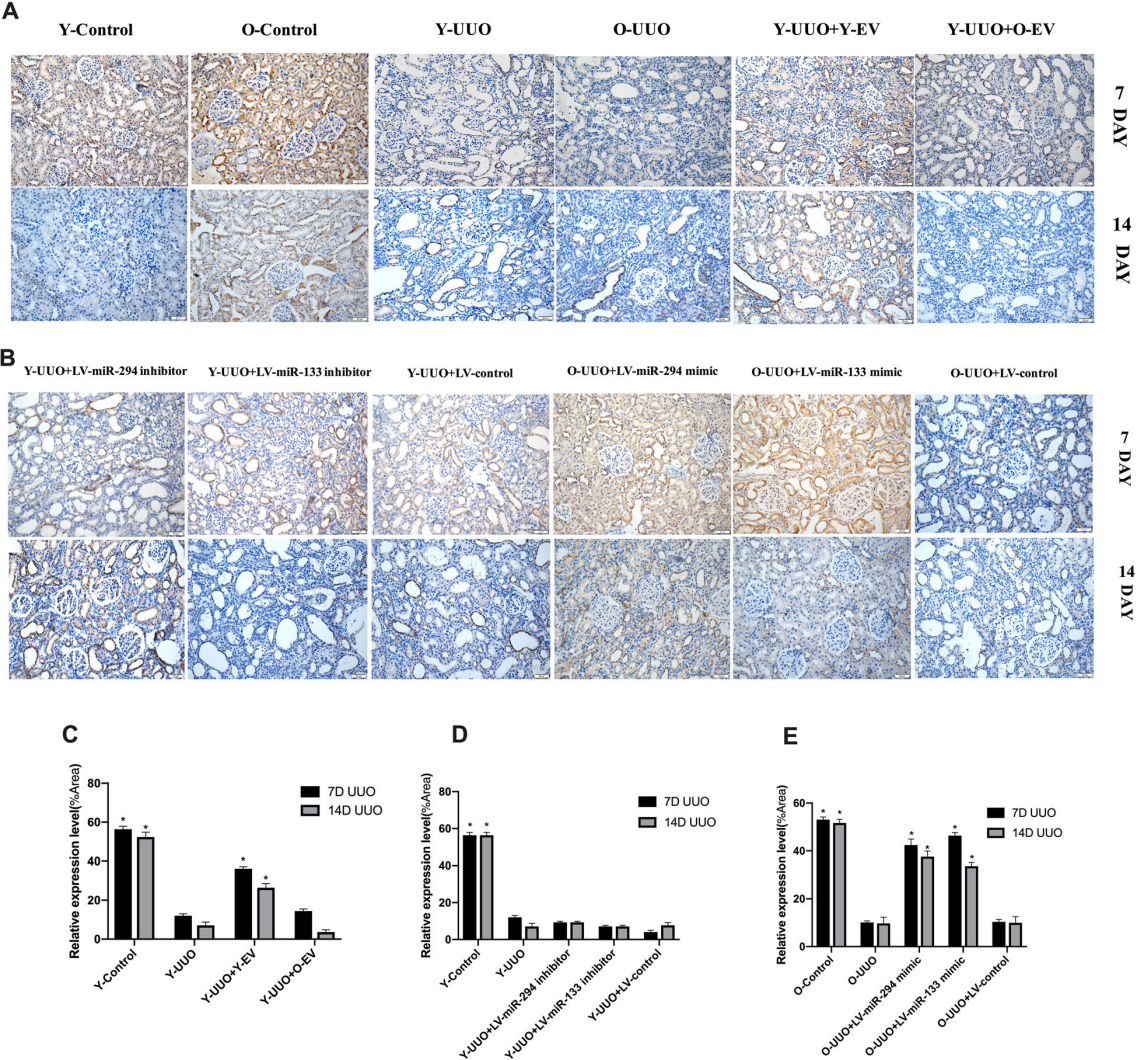
Immunohistochemistry of E-cadherin on the renal histology of kidney sections. **a** The expression of E-cadherin at 7 and 14 days after surgery in control rats and in UUO rats treated with young and old MSC-EVs. **b** The expression of E-cadherin at 7 and 14 days after surgery in control and UUO rats injected with LV-miR-294/133 inhibitor/mimic. **c**–**e** The quantitative analysis of E-cadherin expression in the kidneys. All data are represented as the mean ± SE. The rat number in each group is 5. *p < 0.05 compared with the UUO group

MT staining indicated a time-dependent increase in ECM deposition within the tubulointerstitium in the UUO group, and fibrosis of the tubulointerstitium was more severe on day 14 after UUO surgery. In contrast, Y-MSC-EVs treatment after UUO resulted in an obvious reduction in ECM deposition, but this was not observed in the O-MSC-EVs group (Fig. 4a, c). Moreover, immunohistochemical evaluation of α-SMA, a myofibroblast marker, showed a time-dependent increase in α-SMA-positive areas in the kidneys of UUO rats. However, the administration of Y-MSC-EVs, but not O-MSC-EVs, significantly decreased the α-SMA-positive areas compared to that in UUO rats on days 7 and 14(Fig. 5a, c). Accordingly, with E-cadherin used as a marker of the normal epithelium, E-cadherin-positive areas were markedly reduced in the kidney tissues of UUO rats. E-cadherin expression was increased by the administration of Y-MSC-EVs. These protective effects were not observed in the O-MSC-EVs group (Fig. 6a, c).

**Fig. 7:**
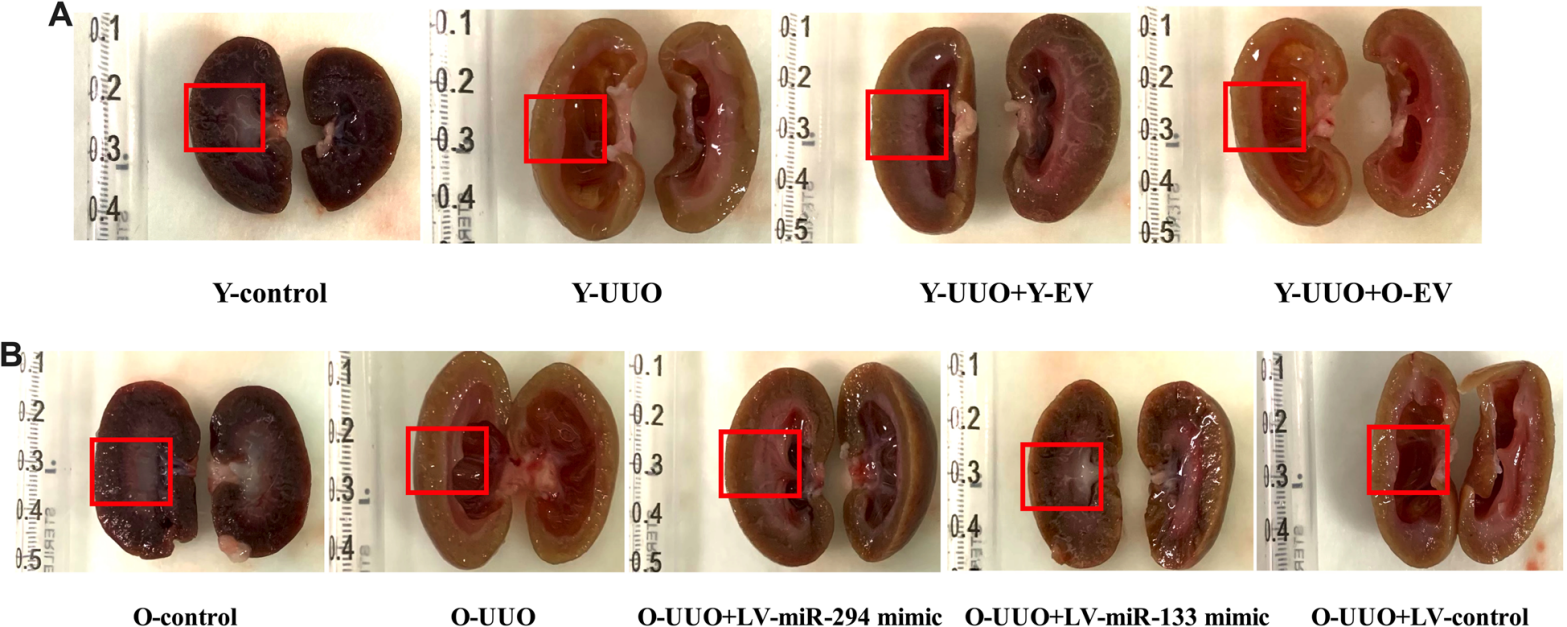
The observation of morphological changes in the kidney. **a** The renal morphology in control young rats and in young UUO rat treated with young and old MSC-EVs at 14 days after surgery. **b** The renal morphology in old control rats and in old UUO rats treated with LV-miR-294/133 mimic at 14 days after surgery. The morphological changes in the renal parenchyma and renal pelvis are marked with a red box

As shown by the morphological observations, the kidneys of UUO rats were obviously hydronephrotic and exhibited a thinner renal parenchyma and dilated renal pelvis on day 14. After injection with Y-MSC-EVs, morphological structural damage was decreased in the O-MSC-EVs group, though no significant changes were observed (Fig. 7a).

### Effects of downregulated miRNAs (miR-294 and miR-133) in MSC-EVs from old rats on UUO-induced CKD

As shown in our previous study [7], miR-294 and miR-133 are significantly decreased in MSC-EVs and sera from old rats. To analyse the potential effects of miR-294 and miR-133 on renal function in old rats, we first investigated whether UUO-induced renal tubulointerstitial fibrosis in pLV-miR-294-mimic- and pLV-miR-133-mimic-treated old rats was decreased by HE staining. On day 7 after UUO operation, obvious renal interstitial broadening and dilated tubules were observed in both young and old rats; more severe renal interstitial broadening was observed on day 14 compared with day 7. However, these pathological features were significantly attenuated in the kidneys of pLV-miR-294-mimic- and pLV-miR-133-mimic-treated old rats (Fig. 3b, e). No significant changes in these pathological features were found in the kidneys of pLV-miR-294-inhibitor- and pLV-miR-133-inhibitor-treated young rats (Fig. 3b, d).

Morphological observations clearly indicated decreased hydronephrosis and thinning of the renal parenchyma, and significantly decreased renal pelvis dilation in the kidneys of pLV-miR-294-mimic- and pLV-miR-133-mimic-treated old rats after UUO surgery compared to old UUO rats on day 14 (Fig. 7b). MT staining showed that the time-dependent increase in ECM deposition within the tubulointerstitium was remarkably improved in pLV-miR-294-mimic- and pLV-miR-133-mimic-treated old rats (Fig. 4b, e). No significant changes in ECM deposition were found in the kidneys of pLV-miR-294-inhibitor- and pLV-miR-133-inhibitor-treated young rats (Fig. 4b, d). Consistent with the results of analysis by MT staining, immunohistochemical staining for α-SMA was lower in the kidneys of pLV-miR-294-mimic- and pLV-miR-133-mimic-treated old UUO rats than the kidneys of vehicle-treated old UUO rats (Fig. 5b, e). Meanwhile, we also found low E-cadherin levels in the kidneys of old UUO rats, and E-cadherin levels were significantly elevated by pLV-miR-294-mimic and pLV-miR-133-mimic treatment (Fig. 6b, e); the improvement in immune-histochemical staining indicated a time-dependent increase in E-cadherin levels on day 14. While, no significant changes were found in the kidneys of pLV-miR-294-inhibitor- and pLV-miR-133-inhibitor-treated young rats with mmunohistochemical staining in α-SMA (Fig. 5b, d) and E-cadherin (Fig. 6b, d).

Biochemical detection showed that while the levels of BUN and UA were increased after UUO operation, these levels were significantly decreased in pLV-miR-294-mimic- and pLV-miR-133-mimic-treated old UUO rats (Fig. 2a, c), no improvement in the level of Scr was detected (Fig. 2e).

### miR-294 and miR-133 overexpression mitigated TGF-β1-mediated EMT

**Fig. 8:**
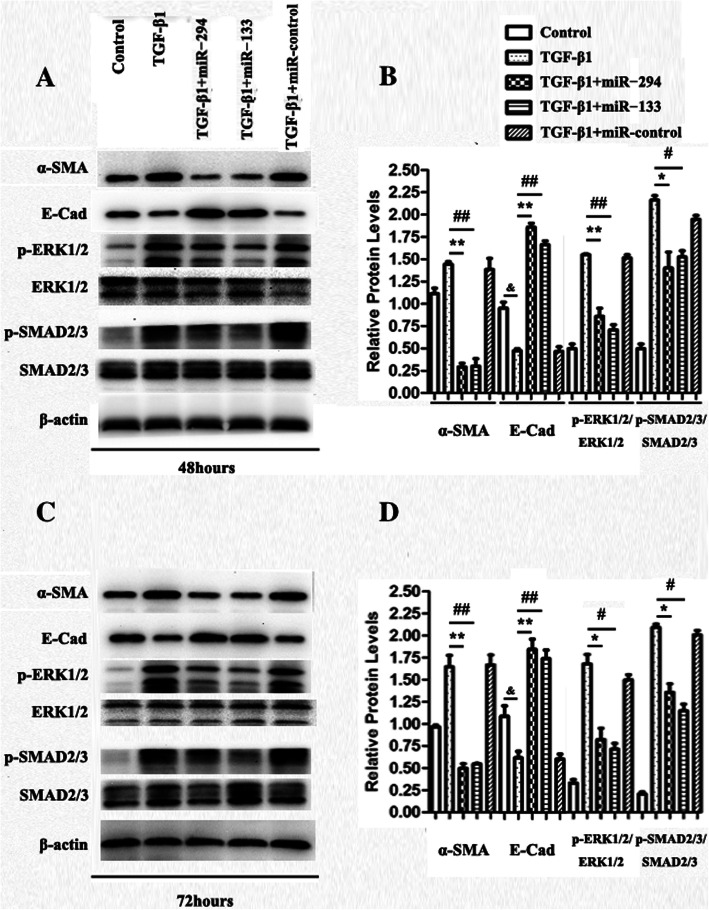
Effect of miRNAs on the inhibitory role of TGF-β1-mediated EMT in HK2 cells

We further explored whether miR-294 and miR-133 can alter the proteins and signalling molecules involved in TGF-β1-mediated EMT in HK2 cells in vitro. The TGF-β1-stimulated group exhibited a time-dependent increase in the protein expression of α-SMA (a marker of fibrosis) as well as a time-dependent decrease in the expression of E-cad, a marker of the normal epithelium (Fig. 8b, d). Recent research demonstrated that ERK activation plays an important role in ECM protein production in EMT [13]; this was also observed in this study by changes in the levels of total and phosphorylated ERK1/2 (Fig. 8a, c). The role of TGF-β1 was indicated by the increased phosphorylation level of SMAD 2/3, a downstream target of the TGF-β1 signalling pathway, in EMT. The obvious increases in α-SMA and the phosphorylation of both SMAD 2/3 and ERK1/2 and the decrease in E-cad were all prevented when miR-294 and miR-133 were overexpressed in HK2 cells (Fig. 8a, b). Also, the prevention effect in EMT of miR-294 and miR-133 indicated a time-dependent increase on 72 h (Fig. 8c, d).

## Discussion

As a global public health problem, CKD causes a high financial burden and impacts the health of affected patients [14]. According to a recent statistical study, the prevalence of CKD in China has reached 10.8%, indicating that approximately 119.5 million people suffer from CKD in China [15]. The glomerular filtration rate (GFR) declines over time after 30 years of age (by approximately 0.75–1.0 ml/min/1.73 m^2^/year) but may increase with diverse diseases (such as hypertension, diabetes and prostatism) over time, leading to a significant increase in the incidence of CKD in elderly individuals [16, 17].

Because of the multipotency, self-renewal, proliferation, immunomodulation and homing of MSCs [18, 19], their differentiation to treat various diseases has received increasing attention, and novel clinical perspectives have been obtained. Stem/progenitor cells, such as BM-MSCs, have been shown to alleviate renal injury, such as AKI and CKD, in both humans and animals [20, 21]. Studies have suggested that MSCs provide significant renal protection by decreasing inflammatory infiltrates, fibrosis and glomerulosclerosis [22]. Recently, researchers found that the restorative effects of MSCs are likely related to their paracrine function and the secretion of several chemokines, cytokines and growth factors. EVs, an important kind of paracrine medium, are defined as a population of vesicles consisting of a mixture of exosomes and shedding vesicles that contain selected mRNA/miRNA profiles and can exchange genetic information between cells. EVs released from stem cells contribute to tissue repair in various animal models of injury [23–25]. Moreover, MSCs release a significant number of EVs with selected expression patterns of mature miRNAs (such as miR-24, miR-29 and miRNA-let7c) that protect against kidney diseases [5, 26, 27].

In fact, the ageing process not only affects the GFR but also decreases the function of MSCs. Senescent MSCs exhibit decreased migration, differentiation potential, immunomodulation and therapeutic effects against injury [28]. The inhibitory effects of MSC-EVs on TGF-β1-mediated EMT is weakened in older rats [7]. Moreover, the miRNAs contained in BM-MSC-EVs can be released into blood circulation where they are converted into circulating miRNAs that possess the ability to change the internal environment, and ultimately impart systemic effects [7, 29, 30]. In future clinical applications, we aim to directly target the circulating miRNAs released from the MSC-EVs. Therefore, we studied whether ageing impacts the restorative effect of EVs from BM-MSCs against CKD and focus on the repair functions of miRNAs downregulated in both MSC-EVs and sera from old rats (miR-294 and miR-133) in renal insufficiency. For young rats, because the contents of miR-294 and miR-133 in MSC and serum were greater than in old rats, we designed a miRNA inhibitor to explore the effects of miR-294 and miR-133 on UUO injury in young rats.

The final common pathological effect of CKD is renal interstitial fibrosis, which is characterised by tubular lesions and the accumulation of ECM [31, 32]. The rat model of UUO is currently regarded as the best animal model of progressive chronic renal tubule interstitial fibrosis [33]. The results of our study in a UUO animal model showed that senescent MSC-EVs derived from old rats exhibited a decreased ability to repair UUO-induced CKD compared to MSC-EVs derived from young rats. Recent studies found that miR-294 can inhibit the TGF-β and GSK3 pathways by regulating TGF-β2 and Gsk3-β [34]. Ectopic transient expression of miR-294 recapitulates developmental signalling and phenotype in cardiomyocytes promoting cell cycle reentry that leads to augmented cardiac function in mice after myocardial infarction [35]. Based on accumulating evidence, miR-133 participates in the proliferation, differentiation, survival, hypertrophic growth and electrical conduction of cardiac cells, which are essential for cardiac fibrosis, cardiac hypertrophy and arrhythmia. Nevertheless, the roles of miR-133 in cardiac remodelling are ambiguous, and the mechanisms are also sophisticated, involving many target genes and signalling pathways, such as RhoA, MAPK and PI3K/Akt [36]. Besides, miR-133 negatively regulates the fibrotic TGF-β/Smad3 pathway as a downstream gene [37], but can also alleviate cardiac fibrosis by preventing SMAD-2 and ERK1/2 phosphorylation [38]. Consistent with these researches, we found that overexpression of miR-294/miR-133 in the serum of old rats exhibited significantly increased prevention of UUO injury, with satisfactorily recovered BUN and UA levels, reduced areas of pathological change and decreased fibrosis. In contrast, our results showed that inhibiting the expression of miR-294/miR-133 had no significant change in young rats after UUO injury, as no obvious further deterioration was observed in young UUO rats in which miR-294/miR-133 were inhibited compared to young UUO rats. This might be because other miRNAs (such as miR-24, miR-29 and miRNA-let7c) play a more important role in renal damage repair than miR-294 and miR-133 in young rats.

The main pathological change in CKD is renal fibrosis, and TGF-β1 is widely regarded as the key cytokine that promotes fibrosis [39]. HK2 is an established human kidney epithelial cell line, and TGF-β1-induced HK2 cells were used to imitate the pathological process of renal fibrosis in vitro in several studies [40, 41]. TGF-β1 induces the transformation of tubular epithelial cells to fibroblast cells, downregulates E-cadherin expression and upregulates SMA expression [42]. Based on these pathological changes and our previous study, we designed a TGF-β1-induced HK2 cell model of EMT to explore the possible mechanism of miR-294/miR-133 in inhibiting renal fibrosis in vitro. We found that following stimulation by TGF-β1, HK2 cells were transformed to a myofibroblast phenotype, resulting in an obvious increase in α-SMA and decrease in E-cadherin protein expression. Interestingly, transfection of the cells with miR-294/miR-133 mimics significantly suppressed α-SMA production and reversed the transformation to a myofibroblast phenotype, which is consistent with the above results from in vivo experiments. Biernacka et al. showed that TGF-β1 relays signal transduction through phosphorylation of the downstream effectors SMAD2/3 [43, 44], and TGF-β1 signal-induced fibrosis in the liver and renal and retinal epithelial cells has been shown to involve the ERK subfamily of stress kinases [45]. Recently, Chen et al. found that cardiac overexpression of miR-133a alleviated cardiac fibrosis by preventing SMAD-2 and ERK1/2 phosphorylation [38]. Thus, in our research, we explored ERK1/2 and SMAD2/3 phosphorylation in TGF-β1-treated HK2 cells with/without miR-294/miR-133 overexpression. The data showed that miR-294/miR-133 mimics could potently inhibit the phosphorylation of SMAD2/3 and ERK1/2 to inhibit TGF-β1-induced EMT in HK2 cells, further restoring renal fibrosis.

## Conclusions

In our study, we found that the inhibitory effects of MSC-EVs against renal fibrosis decreased with age. miR-294/miR-133 in MSC-EVs derived from old rats had an important intervening effect on renal fibrosis. This effect of miR-294/miR-133 may occur through their prevention of SMAD2/3 and ERK1/2 phosphorylation, further influencing renal fibrosis. These findings show that miR-294/miR-133 may be therapeutic in renal fibrosis and related renal dysfunction in elderly individuals.

## Supplementary information

**Additional file 1. **Circulating miRNA expression levels of rats after LV-miRNA injection. A: The relative circulating miR-133 and miR-294 expression in young rats after LV-miR-133/294 inhibitor administration in different time windows. B: The relative circulating miR-133 and miR-294 expression in old rats after LV-miR-133/294 mimic administration in different time windows. *P < 0.05; **P < 0.01; n = 5.

## Data Availability

The datasets used and/or analysed during the current study are available from the corresponding author upon reasonable request.

## Abbreviations

MSCs: Mesenchymal stromal cells
EVs: Extracellular vesicles
MSC-EVs: EVs derived from MSCs
CKD: Chronic kidney disease
UUO: Unilateral ureteral obstruction
TGF-β1: Transforming growth factor-β1
HK2: Human renal proximal tubular epithelial
EMT: Epithelial-mesenchymal transition
ESRD: End-stage renal disease
ECM: Extracellular matrix
miRNA: MicroRNAs
BM-MSCs: Bone marrow mesenchymal stromal cells
PBS: Phosphate buffered saline
FITC: Fluorescein isothiocyanate
PE: Phycoerythrin
SPF: Specific pathogen-free
BUN: Blood urea nitrogen
Scr: Serum creatinine
UA: Uric acid
MT: Masson’s trichrome
HE: Haematoxylin-eosin
DMEM/ F12: Dulbecco’s modified Eagle’s medium/F12
ECL: Chemiluminescence
SMA: Smooth muscle actin
