# Supplementary figures and images for: Protective effect of miRNA-containing extracellular vesicles derived from mesenchymal stromal cells of old rats on renal function in chronic kidney disease
Source: Stem Cell Res Ther. 2020 Jul 8;11:274. doi: 10.1186/s13287-020-01792-7 (PMC7346413; doi:10.1186/s13287-020-01792-7)

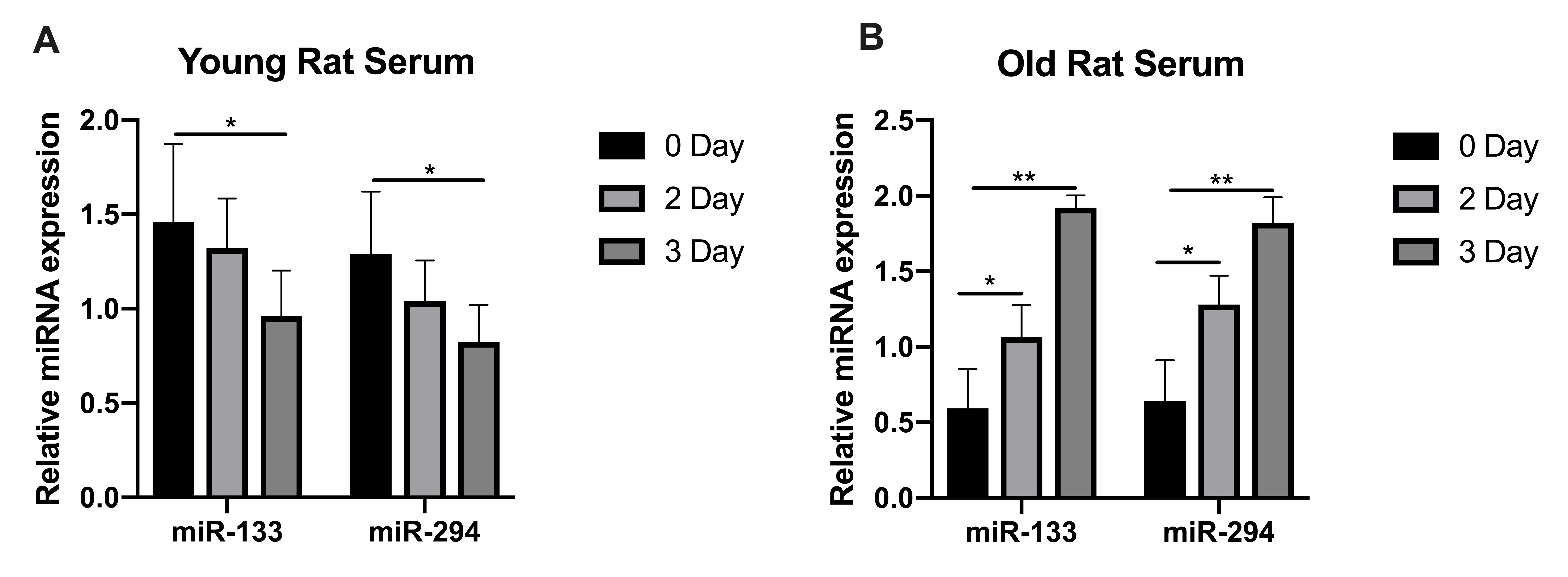

Supplement: Supplementary file 1 — Additional file 1. Circulating miRNA expression levels of rats after LV-miRNA injection. A: The relative circulating miR-133 and miR-294 expression in young rats after LV-miR-133/294 inhibitor administration in different time windows. B: The relative circulating miR-133 and miR-294 expression in old rats after LV-miR-133/294 mimic administration in different time windows. *P < 0.05; **P < 0.01; n = 5. [file 13287_2020_1792_MOESM1_ESM.jpg]
